# Parkinson Phenotype in Aged PINK1-Deficient Mice Is Accompanied by Progressive Mitochondrial Dysfunction in Absence of Neurodegeneration

**DOI:** 10.1371/journal.pone.0005777

**Published:** 2009-06-03

**Authors:** Suzana Gispert, Filomena Ricciardi, Alexander Kurz, Mekhman Azizov, Hans-Hermann Hoepken, Dorothea Becker, Wolfgang Voos, Kristina Leuner, Walter E. Müller, Alexei P. Kudin, Wolfram S. Kunz, Annabelle Zimmermann, Jochen Roeper, Dirk Wenzel, Marina Jendrach, Moisés García-Arencíbia, Javier Fernández-Ruiz, Leslie Huber, Hermann Rohrer, Miguel Barrera, Andreas S. Reichert, Udo Rüb, Amy Chen, Robert L. Nussbaum, Georg Auburger

**Affiliations:** 1 Department of Neurology, University Medical School, Frankfurt am Main, Germany; 2 Institut für Biochemie und Molekularbiologie, University Bonn, Bonn, Germany; 3 Department of Pharmacology, Biocenter Niederursel, University Frankfurt am Main, Frankfurt am Main, Germany; 4 Department of Epileptology, University Bonn, Bonn, Germany; 5 Institute of Neurophysiology, Neuroscience Center, University Frankfurt am Main, Frankfurt am Main, Germany; 6 Max Planck Institute for Biophysical Chemistry, Goettingen, Germany; 7 Department Biochemistry and Molecular Biology and Centro de Investigación Biomédica en Red sobre Enfermedades Neurodegenerativas (CIBERNED), Faculty of Medicine, Complutense University, Madrid, Spain; 8 Max Planck Institute for Brain Research, Frankfurt am Main, Germany; 9 CEF Makromolekulare Komplexe, Mitochondriale Biologie, Fachbereich Medizin, Goethe-Universität Frankfurt am Main, Frankfurt am Main, Germany; 10 Department of Clinical Neuroanatomy, University Med. School, Frankfurt am Main, Germany; 11 National Human Genome Research Institute, National Institutes of Health, Bethesda, Maryland, United States of America; Tokyo Medical and Dental University, Japan

## Abstract

**Background:**

Parkinson's disease (PD) is an adult-onset movement disorder of largely unknown etiology. We have previously shown that loss-of-function mutations of the mitochondrial protein kinase PINK1 (PTEN induced putative kinase 1) cause the recessive PARK6 variant of PD.

**Methodology/Principal Findings:**

Now we generated a PINK1 deficient mouse and observed several novel phenotypes: A progressive reduction of weight and of locomotor activity selectively for spontaneous movements occurred at old age. As in PD, abnormal dopamine levels in the aged nigrostriatal projection accompanied the reduced movements. Possibly in line with the PARK6 syndrome but in contrast to sporadic PD, a reduced lifespan, dysfunction of brainstem and sympathetic nerves, visible aggregates of α-synuclein within Lewy bodies or nigrostriatal neurodegeneration were not present in aged PINK1-deficient mice. However, we demonstrate PINK1 mutant mice to exhibit a progressive reduction in mitochondrial preprotein import correlating with defects of core mitochondrial functions like ATP-generation and respiration. In contrast to the strong effect of PINK1 on mitochondrial dynamics in *Drosophila melanogaster* and in spite of reduced expression of fission factor *Mtp18*, we show reduced fission and increased aggregation of mitochondria only under stress in PINK1-deficient mouse neurons.

**Conclusion:**

Thus, aging *Pink1^−/−^* mice show increasing mitochondrial dysfunction resulting in impaired neural activity similar to PD, in absence of overt neuronal death.

## Introduction

Parkinson's disease (PD) is diagnosed mostly in elderly people by clinical criteria, based on a typical progressive reduction of their spontaneous movement activity in spite of preserved strength and coordination. The clinical deficits reflect progressive neurodegeneration, with initial unspecific symptoms due to pathology in the peripheral autonomic nervous system and in brainstem structures such as the dorsal motor vagal nucleus and the noradrenergic locus coeruleus neurons, and with a later typical movement deficit due to pathology of the dopaminergic neurons projecting from the midbrain substantia nigra to the striatum. Upon autopsy, the diagnostic hallmark of PD is the presence of cytosolic protein aggregates containing α-synuclein within the affected neurons (Lewy bodies). Damage by oxidative stress and mitochondrial dysfunction is documented for affected brain areas, but the molecular events underlying the pathogenesis are unknown. Several neurotoxins and disease genes may cause variants of PD differing in age of onset, manifestations and prognosis, but a common pathway has remained elusive and a routine laboratory diagnosis cannot yet be provided to patients.

A clear genetic link between mitochondria and PD was defined by our identification of loss-of-function mutations in the mitochondrial protein PINK1 as the cause of autosomal recessive early-onset PARK6-linked Parkinsonism, a variant of PD with particularly early onset and mild progression that affects the autonomic and cognitive nervous system less than sporadic PD [1], [2]. PINK1 is a ubiquitously expressed 581 amino-acid protein with a serine-threonine kinase domain and a mitochondrial signal peptide [3]. In skin fibroblasts of 3 PARK6 patients with homozygosity for the G309D-PINK1 mutation, we could demonstrate an impaired activity of the respiratory complex I, an induction of antioxidant defence enzymes and enhanced lipid peroxidation [4], as well as altered α-synuclein mRNA levels [5]. Recombinant PINK1 can protect from apoptosis, modulate the mitochondrial membrane potential [1], localize to mitochondrial cristae [6] and was reported to phosphorylate the mitochondrial chaperone TRAP1 [7] and the mitochondrial protease HTRA2 [8]. In *Drosophila melanogaster*, PINK1 deficiency leads to fragmented mitochondrial cristae, hypersensitivity to oxidative stress, muscle and neuronal degeneration [9], [10] and is rescued by overexpression of parkin and fission factors DRP1 or FIS1, suggesting a physiological role of PINK1 in the dynamics or degradation of mitochondria [11], [12], [13]. Previous studies of *Pink1* knock-out (KO) mouse brain reported (1) elevated susceptibility to H_2_O_2_ or heat-shock with decreased activities of the oxidative-stress vulnerable respiratory complexes as well as aconitase [14], (2) increased calcium levels and vulnerability with subsequent excess ROS production, decreased glucose availability and loss of mitochondrial membrane potential to cause pathological opening of the mitochondrial permeability transition pore [15], (3) reduced synaptic dopamine release and plasticity in the striatum [16], (4) sensitization to activation of group II metabotropic glutamate receptors at corticostriatal synapses [17] and (5) reduced viability of cortical neuron cultures [18]. It remained unclear, however, whether essential mitochondrial functions such as preprotein import and fission/fusion are maintained in the mice, whether the mitochondrial and synaptic dysfunction progress with age to impair movement and behaviour and whether a neurodegenerative process with α-synuclein aggregation is initiated. Since PARK6 is a rare PD variant and patient brain autopsies are still unavailable, the pathological features of PARK6 tissue remain undefined.

To further elucidate the physiological role of PINK1 in mammalian tissue and to gain insight in the disease mechanisms of PARK6, we generated a PINK1 deficient mouse and assessed degrees of mitochondrial dysfunction at old age and the consequences for growth, motor performance, neuronal activity and aggregation/degeneration.

## Results

### Generation of PINK1 deficient mice

**Figure 1:**
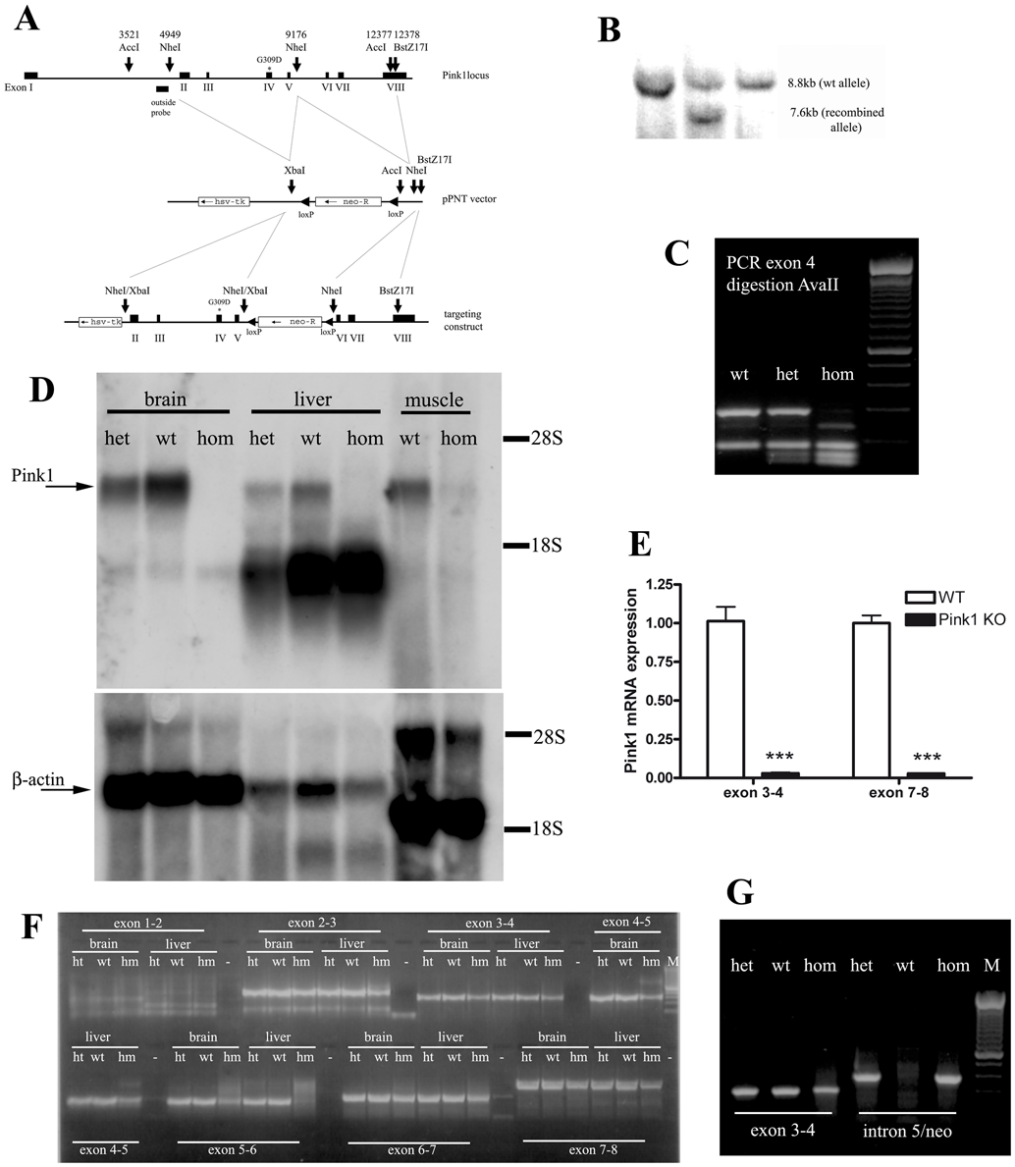
Generation and characterization of the *Pink1* ^−/−^ mouse. (A) Schematic drawing of the targeting strategy. (B) Demonstration of the allele representing the homologous recombination event at the *Pink1* locus by Southern blotting of the outside probe in the embryonal stem cell line, (C) Presence of mutation in homozygous (hom) and heterozygous (het) mouse tail DNA represented by a double band (134+154 basepairs) instead of the wildtype (wt) single band (288 bp) after exon 4 amplification and AvaII restriction. (D) Absence of *Pink1* transcript expression in three tissues of homozygous mutant versus heterozygous and wildtype mice by Northern blotting, using β-actin as control for equal loading and the 18S and 28S ribosomal bands as references for size. One additional cross-reacting band showed intensities not correlating with the mutant genotypes. No residual *Pink1* transcripts of different sizes were detectable, indicating instability and degradation of the mutant mRNA. (E) 97% reduction of *Pink1* mRNA in homozygous KO mice (n = 5) in independent TaqMan systems detecting sequences upstream and downstream from the *Neo* insertion site. (F) Saturation RT-PCR amplification of *Pink1* mRNA sequences containing each splice site, studying brain and liver mRNA from wildtype, heterozygous (ht) and homozygous (hm) mice, demonstrating abnormalities in homozygous tissues with additional large bands for the exon 4–5 splicing boundary and smears of abnormally large size for the exon 5–6 boundary. (G) Saturation RT-PCR with primers from *Pink1* intron 5 and the *Neo* selection marker demonstrating the pathological presence of intronic and *Neo* sequences in *Pink1* mRNA from mutant (ht and hm) mouse samples.

The pathogenic G309D-PINK1 mutation of our PARK6 patients was inserted into the orthologous mouse locus, using a neomycin (*Neo*) selection marker, flanked by loxP sites, engineered in intron V (Fig. 1A). The targeting vector was designed to allow a substitution of the G309D mutation, with minimal disruption of the *Pink1* gene. Homologous recombination in embryonal stem cells was demonstrated by Southern blotting with an outside probe (Fig. 1B) and presence of the mutation in tail DNA by PCR amplification and restriction (Fig 1C). However, Northern blots with a probe covering *Pink1* exons III to VI demonstrated no full-length *Pink1* expression in the mice carrying this allele (Fig. 1D), suggesting interference with gene expression by the presence of *Neo*. The expression levels of the mutant *Pink1* mRNA were reduced by a factor of 14 when analyzed by the Affymetrix transcriptome microarray and by a factor of 35 in quantitative real time RT-PCR (qPCR). To assess whether the intense smaller band detected by Northern blot represents a *Pink1* isoform, qPCR analysis of *Pink1* mRNA sequences on both sides of the *Neo* insertion site were performed and showed a 97% reduction in homozygous mutant brain tissue in both cases, indicating that the smaller band in Northern is due to unspecific cross-hybridization (Fig. 1E). Saturation reverse transcriptase (RT) PCR of each splicing boundary in brain and liver mRNA specifically detected normally spliced amplicons in wildtype (wt) and heterozygous (ht), but in homozygous tissues (hm) a smear of abnormally large amplification products was found for the exon 5–6 boundary suggesting the presence of intron 5 including the *Neo* selection marker (Fig. 1F). Additional saturation RT-PCR experiments confirmed the presence of *Pink1* intron 5 and *Neo* sequences in the residual *Pink1* mRNA and demonstrated the existence of smears of abnormally sized amplification fragments in homozygous tissue (Fig. 1G), suggesting that a *Pink1* mRNA with excessive size is unstable and degraded to about 3% residual expression levels. Endogenous PINK1 protein cannot be detected reliably, probably due to very low expression levels [19]. Attempts to concentrate endogenous PINK1 protein through degradation inhibition and immunoprecipitation were promising in HEK293 but inconclusive in tissues, because the antibodies cross-reacted with more abundant proteins. Thus, a demonstration of the PINK1 protein deficiency was not possible. Since a loss-of-function of PINK1 is the cause of PARK6, these mice with a 97% knock-down of *Pink1* transcript and a loss-of-function mutation in the remaining mRNA represent a useful disease model. Their quality is similar to the previously reported *Pink1* knock-out mice generated by germline deletion of exons 4–7 resulting in an abnormally sized transcript which is probably degraded since no expression was detectable in Northern blots [16]. The other published *Pink1* knock-out mouse was generated by deletion from exon 2 to 5 and is predicted to produce an abnormally sized and unstable *Pink1* transcript, but no Northern or qPCR data are available and its sole expression characterization was through Western blotting in spite of the known problems to detect the endogenous PINK1 protein in tissues [18]. Although residual amounts of abnormally spliced *Pink1* transcript would exist in all these *Pink1* mutant mouse lines and it could be speculated that residual amounts of PINK1 protein with abnormal 3′ sequences are relevant, it is highly probable that mouse phenotypes would result from the engineered deficiency rather than from sequence abnormalities of residual PINK1.

### Progressive reduction of weight and of spontaneous locomotor activity

While all PD variants show the typical deficit of spontaneous locomotor activity from deficient dopaminergic nigrostriatal signaling, early disease stages of non-inherited PD and some inherited PD variants are accompanied by unspecific non-motor symptoms such as hyperhidrosis (excessive sweating) caused by dysfunctional sympathetic nerves [20] as well as altered stress responses due to brainstem noradrenergic neuron pathology [21]. Although there is an ongoing debate which assays are best to quantify these alterations, we made an effort to assess the functional consequences of PINK1 deficiency for both motor and non-motor nervous system deficits as well as growth and survival of the whole organism, using homozygous mutant and wildtype cohorts in pure 129/SvEv background, both derived from the founding heterozygous animals.

**Figure 2:**
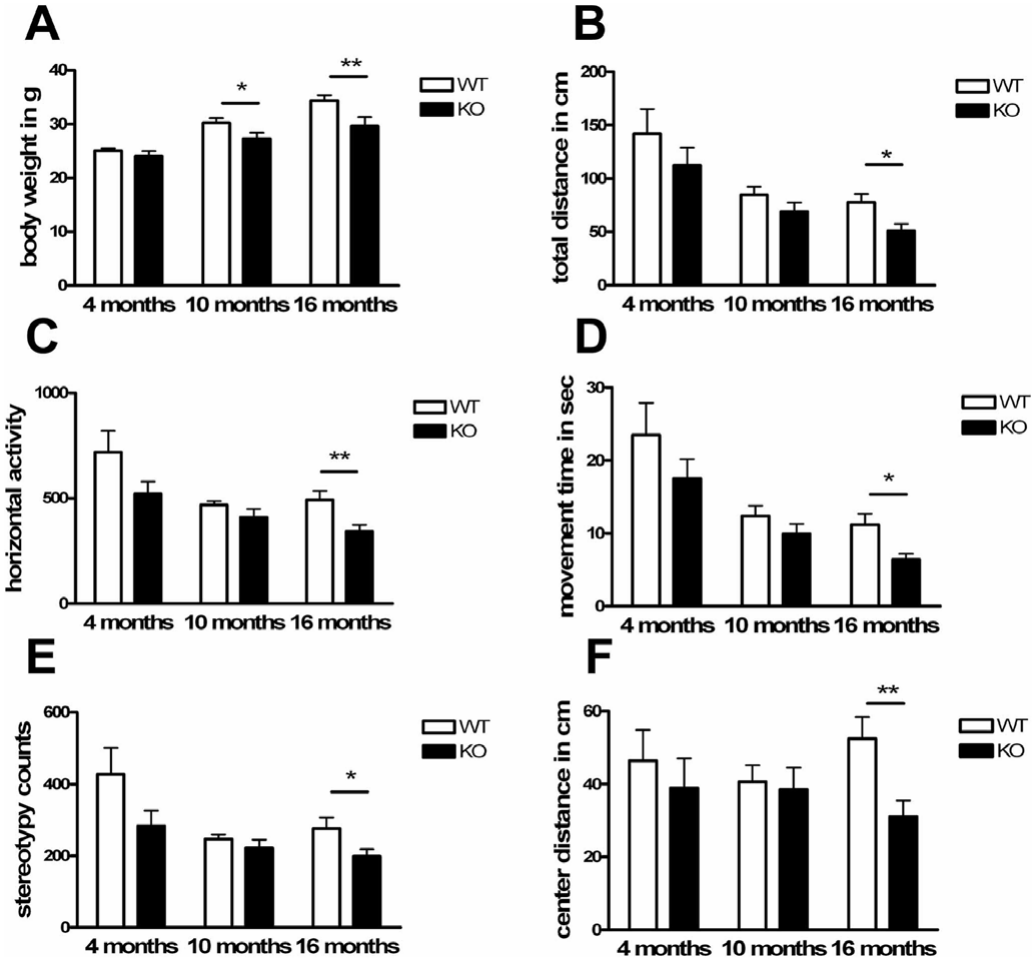
Progressive phenotype upon visual inspection of *Pink1* ^−/−^ mice. Knock-out (KO) versus wildtype (WT) mice showed (A) a consistent and progressive reduction of body weight from middle age and (B–F) a reduction of spontaneous movement, significant for the open-field parameters (B) total distance, (C) horizontal activity, (D) movement time, (E) stereotypy counts and (F) center distance at advanced age.

Already at 1 year of age, the body weight of *Pink1* ^−/−^ mice showed a significant reduction in average weight of up to 19% (Fig. 2A), a finding which may reflect impaired bioenergetics and mitochondrial dysfunction. This weight reduction occurred although the animals were unlimited in their movement range and certainly able to reach the food, although formal assessment of food intake and metabolism was not performed. A significant reduction of locomotor activity was not demonstrable until the age of 16 months, possibly because wildtype mice from the 129/SvEv strain are known for low motor activity. Impairment of spontaneous movement became significant for several parameters in open field analysis (Fig. 2B–F) of 16 animals of the first homozygous generation. The measures for total distance (TOTDIST), horizontal activity (HACTV), movement time (MOVTIME), stereotypy count (STRCNT) and center distance (CTRDIST) were all decreased by about 30% (p<0.05), while measures for strength (Fig. S1), coordination (Fig. S2) and anxiety (Fig. S3) remained normal. The significant deficit in spontaneous movement activity was independently reproduced in subsequent progeny of the first cohort (8 animals per group at age 24 months, 40–55% reduction with p<0.05 for each of the above parameters). Such selective movement impairment is usually caused by reduced dopaminergic signalling in the striatum, but this assumption can be difficult to prove by behaviour tests since pharmacological substitution of dopamine by L-Dopa or apomorphine would lead to hyperactivity both in mutant and wildtype mice.

The movement deficit was not severe enough to reduce the lifespan of PINK1 deficient mice (Fig. S4). Non-motor nervous dysfunction was not detectable: Increased sweating was not observed in aged *Pink1* ^−/−^ mice even under pharmacological provocation (Fig. S5). Assessment of startle reflexes to acoustic stimuli, which represent brainstem functions modulated by the noradrenergic locus coeruleus neurons, did not reveal any impairment (Fig. S6). Thus, the early features of sympathetic affection and brainstem locus coeruleus pathology typically documented in early stage sporadic PD were not observed in our *Pink1* ^−/−^ mice, suggesting that the effect of PINK1 deficiency in mouse does not reach this stage or has different tissue specificity. In contrast, the movement deficit with selectivity for spontaneous locomotor activity mirrors the diagnostic features of PD.

### Deficiency of striatal dopamine at advanced age, in spite of absent neurodegeneration in the dopaminergic projection from the substantia nigra to the striatum

**Figure 3:**
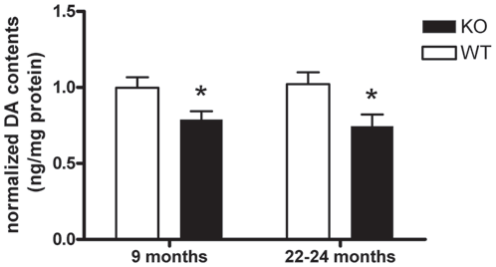
Aged *Pink1* ^−/−^ brain contains decreased dopamine amount. HPLC analysis of *Pink1* ^−/−^ striatal homogenates detected a significant reduction of dopamine (DA) at 9 and 22–24 months of age (n = 8 for each group).

**Figure 4:**
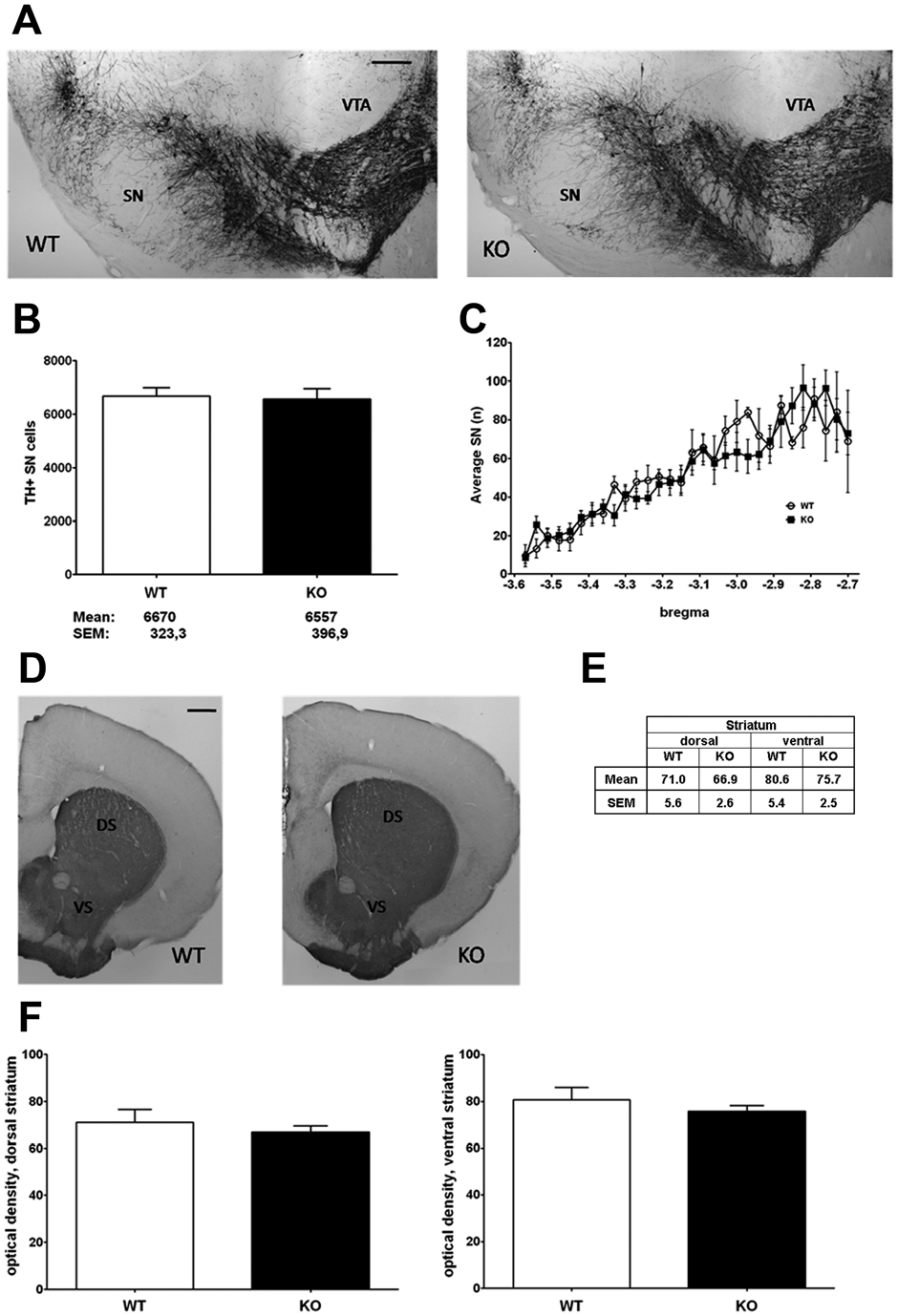
Aged *Pink1* ^−/−^ brain does not display the typical signs of Parkinsonian neurodegeneration. (A–C) Stereological counts of dopaminergic neurons in midbrain tissue: (A) Tyrosine hydroxylase (TH) immunostaining of wildtype (WT) and *Pink1* ^−/−^ (KO) substantia nigra (SN) and ventral tegmental area (VTA) (scale bar, 200 µm). (B) Nonbiased stereological quantification of TH-immunopositive cells from WT (n = 5) and KO (n = 6) SN. The mean neuron count differed only by 113 cells. (C) Average number of unbiased sampled TH-immunopositive SN neurons in 30 serial midbrain sections, covering the caudo-rostral axis (bregma −3.8 to −3.2) in WT (n = 4) and KO (n = 6) mice. Note that there are no significant differences between the two graphs. (D–F) Optical density of dopaminergic nerve terminals in dorsal and ventral striatum: (D) TH immunostaining of striatal sections from WT and KO mice (scale bar, 500 µm). (E, F) Optical density quantification of TH immunostaining density of dorsal (DS) and ventral striatum (VS) from WT (n = 5) and KO (n = 6) mice. There were no significant differences in dorsal (mean WT 71.0, SEM 5.6; KO 66.9, SEM 2.6) and ventral striatum (WT 80.6, SEM 5.4; KO 75.7, SEM 2.5).

To investigate striatal dopaminergic signalling as the likely cause of a selective deficit in spontaneous movement activity, high pressure liquid chromatography (HPLC) studies of striatal tissue dopamine levels were performed in PINK1 deficient mice. At ages 9 and 22–24 months, a significant reduction of dopamine content was observed (Fig. 3). These findings extend a previous report of altered dopamine release and synaptic plasticity in striatal slice cultures from 3 months old *Pink1* knock-out mice [16]. To determine whether the onset of neurodegeneration underlies this dopamine deficit at advanced ages, we performed a systematic histological evaluation of neuron populations through Nissl staining, of myelinated fibre tracts through Klüver-Barrera counterstaining, and of astrogliosis through GFAP immunohistochemistry in sections from the lower brainstem to the frontal cortex taken at regular intervals. In agreement with the behaviour tests excluding ataxia, peripheral paralysis, autonomic and acoustic dysfunction, no obvious structural pathology was found in the cerebellum, the brainstem and the cerebrum at large. In view of the preferential susceptibility of nerve cells and nerve terminals of the dopaminergic pathway from the substantia nigra (SN) to the striatum to the disease process of PD and to mitochondrial complex I toxins such as MPTP and rotenone, we focussed on a thorough quantification of this projection with morphological and biochemical criteria. After specific detection of these cells with tyrosine hydroxylase (TH) immunohistochemistry in brain tissue at age 18 months, the neuronal cell bodies were counted by unbiased stereology in 30 serial sections covering the whole SN. No loss of dopaminergic neurons was apparent in the PINK1 deficient tissue (Fig. 4A–C). Furthermore, no reduction in the optical density of TH-positive nerve terminals in the striatum could be documented (Fig. 4D–F). Thus, the earlier report of unchanged number of TH-immunoreactivity in the nigrostriatal pathway in *Pink1* knock-out mice until age 9 months [16] is confirmed and extended to the age of 18 months in our mice. Thus, the data suggest that the reduced locomotor activity in aged PINK1 deficient mice might be caused by impaired dopaminergic neurotransmission, but is not due to neurodegeneration in the nigrostriatal projection.

### Absence of Lewy bodies from brainstem and substantia nigra neurons, which are known for their high vulnerability in PD

To assess whether the protein aggregation typically seen in neurons affected by PD is a feature of aged *Pink1^−/−^* brain, the subcellular localization and expression of α-synuclein was studied by immunohistochemistry, immunoblots and qPCR. A lightmicroscopical inspection of brain sections at regular intervals throughout the brain stained with α-synuclein immunohistochemistry did not reveal any visible cytoplasmic aggregates. In particular, the substantia nigra was free of aggregates. Only in the lower brainstem, enhanced α-synuclein immunoreactivity within neurons in somatodendritic distribution and throughout the neuropil was observed in independent analyses of the dorsal motor nucleus of the vagus (Fig. S7). Enhanced expression and somatodendritic distribution of α-synuclein correlate with aggregation in several mouse mutants [22]. Therefore, quantification of the α-synuclein expression was performed with immunoblots and qPCR. Both in midbrain and in brainstem a slight increase in α-synuclein protein levels did not reach significance. However, α-synuclein mRNA levels in both regions were significantly reduced (Fig. S8). These findings might indicate impaired degradation and reduced re-synthesis of α-synuclein and suggest an emerging protein clearance pathology.

### Mitochondrial dysfunction is progressive from early ages

In order to understand how PINK1 deficiency results in reduced growth, spontaneous locomotion and striatal dopamine levels, we studied mitochondrial function in detail. Recombinant PINK1 was previously localized to mitochondrial cristae [6] or outer membrane [19], impaired respiratory activity and oxidative stress are detectable in patient tissues, and PINK1 deficiency phenotypes in *Drosophila melanogaster* mutants are rescued by factors affecting mitochondrial dynamics [11], [12], [13]. Since PINK1-related mitochondrial dysfunction was previously documented not only in tissues affected by Parkinsonian neurodegeneration, but also in skeletal muscle [9], [10] and skin fibroblasts [4], it probably represents an early mutation consequence and cannot be the sole cause for tissue-specific disease vulnerability; we therefore extended our study to liver and dissociated cells from whole brain.

**Figure 5:**
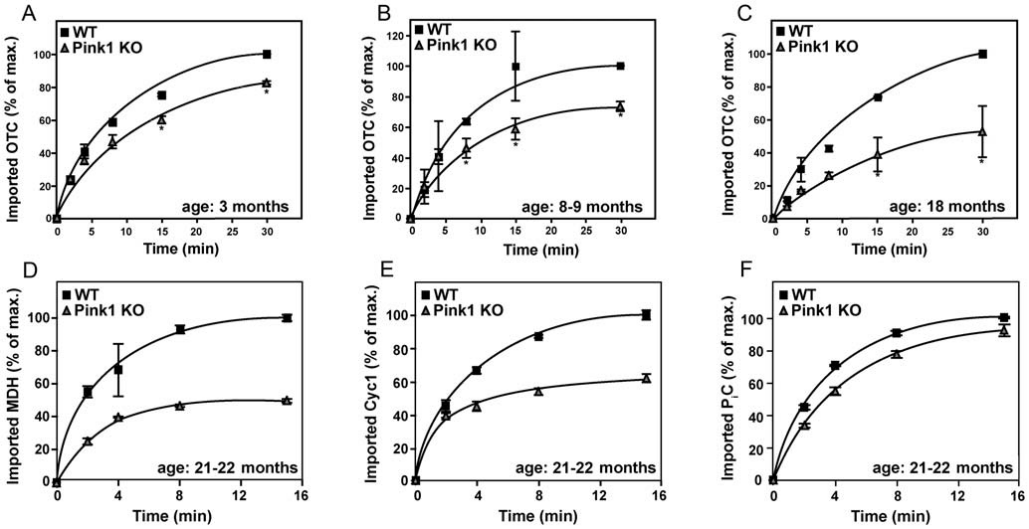
Progressive deficit in mitochondrial preprotein import. The reduction of mitochondrial import of (OTC) precursor protein in *Pink1* ^−/−^ mouse liver mitochondria (n = 2–4 animals for each group and age) was increasingly impaired with advanced age (A–C). Import of the matrix and inner membrane markers human malate dehydrogenase (MDH) (D) and cytochrome *c1* (E) was reduced, while import of the inner membrane phosphate carrier protein (P_i_C) as marker of an alternative translocation pathway (F) was not affected in old animals.

Mitochondrial functions are dependent on a biogenesis process that delivers proteins by a specific protein import reaction, comprising three separate biochemical processes (recognition, translocation to mitochondrial subcompartments and processing). In view of the report on PINK1 phosphorylating the mitochondrial chaperone TRAP1 [7] and the role of chaperones in mitochondrial preprotein import, we assessed the residual translocation competence of PINK1 mutant mitochondria by *in vitro* import reactions using mitochondria isolated from liver tissue obtained from *Pink1* ^−/−^ mice at different ages. To quantify import, the necessary high number and purity of mitochondria could be obtained from the homogeneous and mitochondria-rich liver tissue, but not from brain. Mitochondria were incubated with different radiolabeled precursor proteins destined for the matrix compartment and the inner membrane. The translocation of the rat ornithine transcarbamylase (OTC) precursor protein to its protease-protected localization in the mitochondrial matrix showed significant age-related progressive defects. Already at an age of 3 months, a small but significant reduction of the import efficiency of OTC could be observed. This import defect became more severe at an age of 6 months and was reduced by nearly 50% by the age of 18 months in comparison to age-matched wildtype mitochondria (Fig. 5A–C). A similar import defect was observed with the precursor of the human matrix malate dehydrogenase (MDH), but also with cytochrome *c1*, an inner membrane respiratory chain component (Fig. 5D–E). In contrast, the import of the inner membrane phosphate carrier protein (P_i_C), which utilizes a different translocation pathway [23] showed only minor reductions (Fig. 5F), correlating with only minor reductions of the inner membrane potential (Δψm) in liver mitochondria (data not shown).

**Figure 6:**
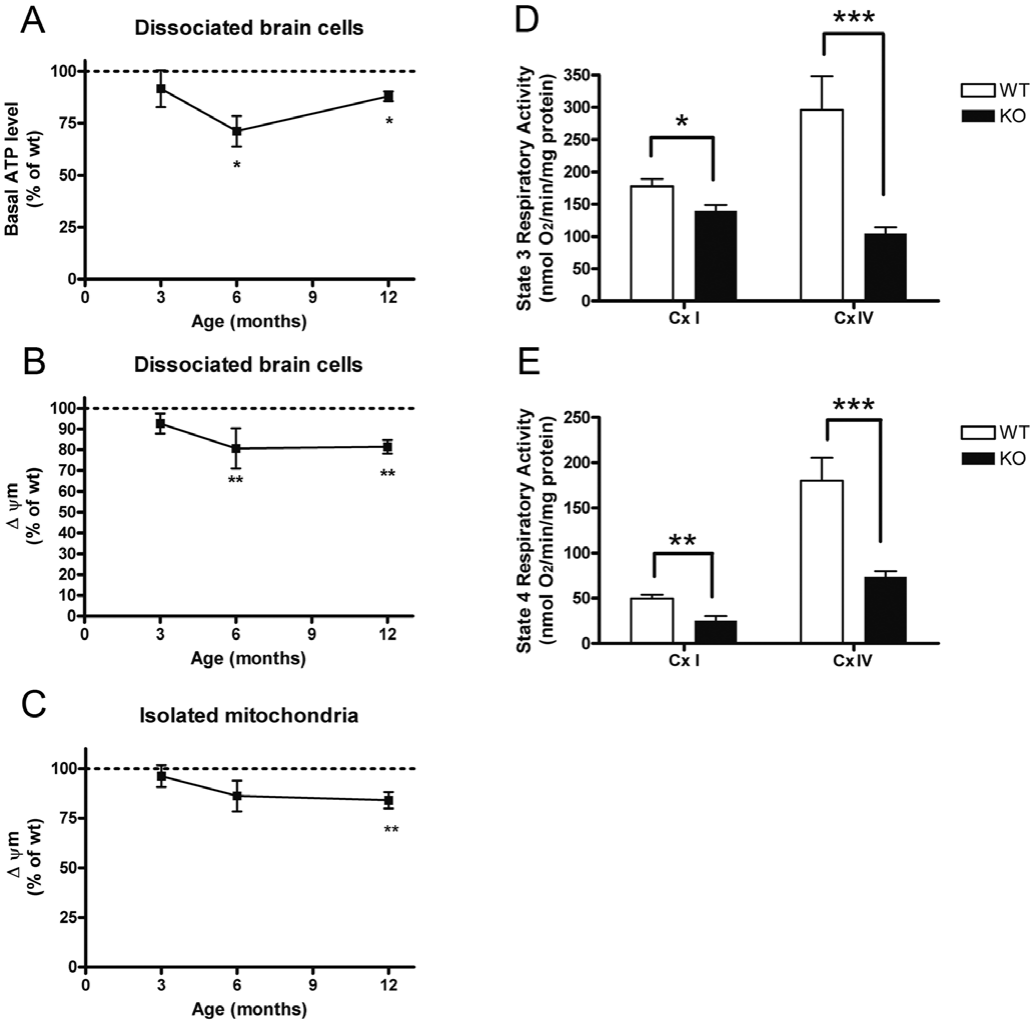
Progressive pathology of mitochondrial bioenergetic function in *Pink1* ^−/−^ brain. Reduced basal ATP level in *Pink1* ^−/−^ dissociated cells (A), reduction of basal mitochondrial membrane potential Δψm in (B) dissociated cells and in (C) isolated mitochondria from *Pink1* ^−/−^ versus wildtype brain, as well as reduction of respiratory activities for complexes I+III+IV (Cx I) and IV (Cx IV) (D,E) substantiate a mitochondrial dysfunction at old age.

The primary function of mitochondria consists of providing cellular ATP by the process of oxidative phosphorylation. ATP levels under basal conditions were significantly reduced in dissociated *Pink1* ^−/−^ mouse neurons (Fig. 6A), similar to what was seen previously in *Pink1* ^−/−^ *Drosophila melanogaster* muscle [9], [10]. The mitochondrial membrane potential (Δψm) of dissociated *Pink1* ^−/−^ neuron-glia cultures and of isolated *Pink1* ^−/−^ brain mitochondria showed a significant and progressive reduction even under basal conditions, without the stress conditions published for transfected SH-SY5Y cells [1] (by 19.4% and 13.8%, respectively, at 6 months and 18.6% and 16.0% at 12 months, Fig. 6B–C). As a potential correlate of the Δψm reduction at old age, we investigated the respiratory complex activity of purified mitochondria from 18 months old brain tissue. Reduced activity was observed for the respiration with glutamate+malate using respiratory complexes I+III+IV, as previously reported [14], but also for TMPD+ascorbate using respiratory complex IV (Fig. 6D–E).

The results indicate that a novel and early deficit in liver mitochondrial preprotein import and a progressive impairment of brain mitochondrial bioenergetics are prominent phenomena in *Pink1* ^−/−^ mice and may be important contributors to the weight and activity phenotypes.

### Impairment of mitochondrial fission under stress and *Mtp18* reduction in *Pink1* ^−/−^neurons

**Figure 7:**
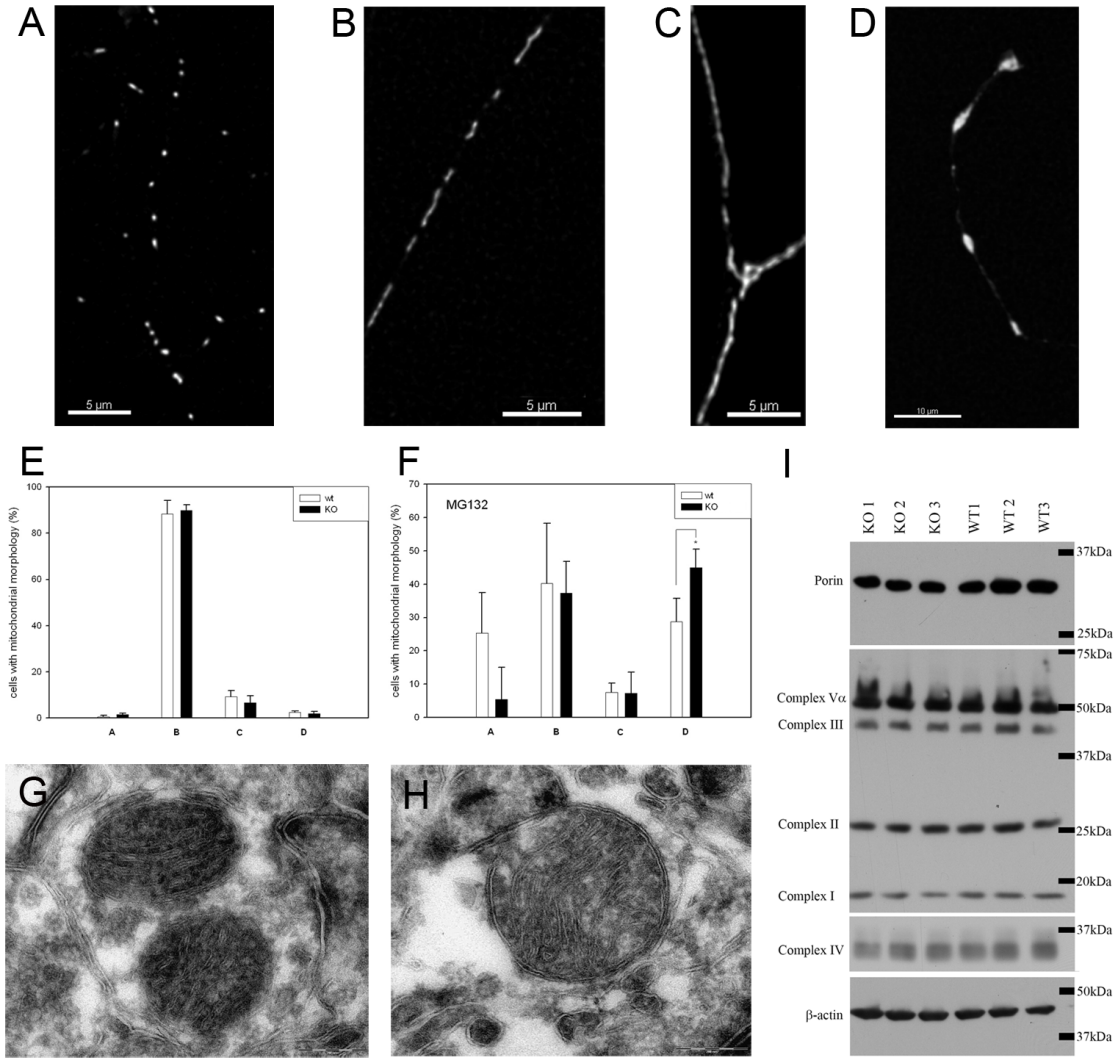
Early stress-induced deficit of mitochondrial fission in *Pink1* ^−/−^ neurons. Postnatal primary cortical neuron cultures stained with Mitotracker were analyzed by quantification of mitochondrial morphotypes (A = fragmented, B = tubular, C = long tubular/network, D = aggregated) under unstressed conditions (E) or after proteasomal stress with MG132 (F). Without stress, there were no differences in the mitochondrial morphology between wildtype and *Pink1* ^−/−^ neuronal cells. Under stress, fewer fragmented mitochondria and significantly more aggregated mitochondria were detected in *Pink1* ^−/−^ neurons, indicating a loss of stress-induced fragmentation in *Pink1* ^−/−^. Electron microscopy failed to demonstrate a loss of cristae or other conspicuous structural pathology, as seen in two representative sections from of *Pink1* ^−/−^ brain at age 18 months (G, H). Immunoblots of marker proteins residing in inner (complex I–V) or outer mitochondrial membranes (porin) from 3 different mice of age 21 months did not show a proliferation of mitochondrial mass or an altered inner versus outer membrane ratio (I).

Since several investigators have reported PINK1 to act as mitochondrial fission factor in *Drosophila melanogaster* [11], [12], [13], we analyzed whether fission impairment accompanies the mitochondrial dysfunction in PINK1 deficient mice, studying primary neuron culture and aged brain tissue with fluorescent microscopy and electron microscopy, respectively. Upon fluorescent staining with Mitotracker, the mitochondria of postnatal cortical primary *Pink1* ^−/−^ neurons showed a normal distribution of morphotypes (Fig. 7A–E). Under conditions of proteasomal stress, the amount of fragmented mitochondria appeared reduced while the aggregated mitochondria in a perinuclear localization were significantly increased (7F), consistent with a role of PINK1 for stress-induced fission. Upon electron microscopy of aged *Pink1* ^−/−^ brain, the loss of cristae reported for *Drosophila melanogaster* could not be demonstrated (Fig. 7G–H). To assess whether a possible alteration of mitochondrial biogenesis or degradation results in altered levels of mitochondria, we quantified marker proteins of the mitochondrial inner and outer membrane from aged *Pink1* ^−/−^ brain with immunoblots. No significant differences were observed (Fig. 7I). Since the relative normality of mitochondrial dynamics in mouse brain contrasts with a strong phenotype in *Drosophila melanogaster* after PINK1 loss, we speculated that compensatory mechanisms in mammalian tissues exist. Assessing the expression levels of other factors known to modulate mitochondrial dynamics, no compensatory changes were found. However, the significantly reduced transcript level of the recently described fission factor MTP18, consistent from 2 days to 18 months of age (Table 1), substantiated the subtle impairment of mitochondrial fission. In spite of the Δψm reduction in aged *Pink1* ^−/−^ brain, the ratio among the various processed isoforms of the fusion factor OPA1 remained normal (Fig. S9). Thus, mitochondrial morphology appears to be relatively balanced in PINK1 deficient mammalian tissue, unless cell stress is present.

**Table 1:** Expression levels of mitochondrial fission and fusion factors in *Pink1* ^−/−^ tissue.

Taqman® assay	mitochondrial function	gene symbol	liver 2 days	brain 2 days	liver 18 months	brain 18 months
Mm00612599_m1	fusion	MfnI	ns	ns	ns	ns
Mm00500129_m1	fusion	MfnII	ns	ns	ns	ns
Mm00453879_m1	fusion	OpaI	ns	ns	ns	ns
Mm00481580_m1	fission	FisI	ns	ns	ns	ns
Mm01342903_m1	fission	DrpI	ns	ns	ns	ns
Mm00466042_m1	fission	Mtp18	red. 25%; p = 0.04	red. 10%; p = 0.09	red. 30%; p = 0.0002	ns

A selective reduction of the fission factor *Mtp18* mRNA levels in qPCR analysis was consistently significant at ages 2 days and 18 months for the particularly mitochondria-rich and homogeneous liver tissue, while the effect missed significance for the mixed tissue brain.

red. = reduced by factor; ns = non-significant.

## Discussion

In this study, we generated PINK1 deficient mice to elucidate the physiological function of PINK1 and to model human PARK6, a particularly interesting variant of PD in view of its monogenic loss-of-function mechanism and its putative mitochondrial pathogenesis. Here, for the first time we report a phenotype of motor impairment at old age with characteristics similar to PD, together with evidence of dopaminergic synapse dysfunction and prominent mitochondrial dysfunction, which is detectable early under stress through impaired fission and appears also in tissues unaffected by PD such as liver, but which worsens with increasing age just like the motor deficit and includes a prominent deficit of preprotein import. Related mechanisms of mitochondrial dysfunction are likely also important in the more frequent sporadic form of PD in elderly people, since an increased frequency of somatic mitochondrial DNA deletions was recently observed in midbrain tissue of such patients, with consequent respiratory chain deficiency and probably oxidative stress [24].

### PINK1 deficiency and mitochondrial dysfunction

Mitochondria are important mediators of neural function and synaptic plasticity [25]. Our observation that PINK1 deficiency in mouse brain leads to a combined impairment of activity of respiratory complex I+III+IV and II+III+IV, mitochondrial membrane potential Δψm and ATP generation at old age are consistent with a defect in mitochondrial bioenergetic function. They extend previous reports about reduced Δψm and elevated oxidative stress in human dopaminergic neuron cultures with *Pink1* knock-down [18] and about reduced mitochondrial respiration and increased sensitivity to oxidative stress in *Pink1* knock-out mice and *Drosophila melanogaster* [14], [26]. In addition, our investigation of liver tissue revealed a mitochondrial import defect, which primarily affected preproteins targeted to the matrix compartment, indicating that the reduction of translocation efficiency was not solely caused by a reduced inner membrane potential. Due to the basic relevance of preprotein import for mitochondrial biogenesis, the observed defects could significantly contribute to the respiration deficiencies in PINK1 mutant mitochondria. Although the chain of events leading to this respiration deficit is currently not understood, the findings constitute a robust assay for the progressive pathology induced by PINK1 deficiency. While all data agree that PINK1 exerts an important, but possibly secondary effect on mitochondrial bioenergetics, the function of PINK1 for mitochondrial dynamics, fragmentation and degradation appears less prominent in mouse tissue. In PINK1 deficient *Drosophila melanogaster*, several independent groups reported clear-cut pathology of mitochondrial cristae and elongation of mitochondrial networks as evidence for a PINK1 role in fission dynamics [11], [12], [13]. In contrast, the study of mammalian PINK1 deficient tissue produced controversial findings: human cells with knocked-down or mutant *Pink1* showed either a paradoxical increase in fragmented mitochondria [27] or proliferation of mitochondria with abnormal morphology [18]. PINK1 deficient mice showed normal mitochondrial mass and structural integrity with a marginal increase of mitochondrial size [14]. Our observation of selective reduction of *Mtp18* transcript in PINK1 deficient tissue at different ages represents evidence of a subtle fission deficit and identifies a candidate molecule in the PINK1 pathway. MTP18 was recently described as a transcriptionally regulated target of phosphatidylinositol 3-kinase signalling that is localized within mitochondrial membranes and is thought to provide the limiting step during the fission process of the inner membrane, in parallel to the action of FIS1 at the outer membrane [28], [29]. While our data indicate mitochondria of PINK1 deficient mice to have normal morphology and mass in aged brain, they did show enhanced perinuclear aggregation after proteasomal stress, suggesting that PINK1 plays a role in mitochondrial fission and/or autophagic degradation (mitophagy). In PINK1-deficient mouse brain tissue under physiological conditions, this function can apparently be compensated by downstream components of the same pathway, while very recent data showed a stable *Pink1* knock-down in human SH-SY5Y neuroblastoma cell culture to induce mitochondrial fragmentation and autophagy [30]. PINK1 has been shown to act upstream of the ubiquitin ligase parkin [9], [10], which is required for autophagic clearance of damaged mitochondria [31]. PINK1 was found to reside in the mitochondrial outer membrane with its kinase domain facing the cytosol [19] and was claimed to phosphorylate cytosolic parkin, mediating the translocation of parkin to mitochondria [32]. Thus, PINK1 might be a sensor of mitochondrial or cellular stress, and might trigger the initiation of mitophagy through parkin activation [33]. In accordance with this hypothesis, the loss of PINK1 would become noticeable under stress through a failed activation of mitophagy, resulting in the observed perinuclear accumulation of mitochondria. Alternative evidence indicates that PINK1 deficiency results primarily in a bioenergetic deficit and oxidative stress and that damaged mitochondrial fragments are removed and degraded in a compensatory effort [30]. While elucidation of the precise actions of PINK1 has to await the identification of PINK1-phosphorylation substrates, in *Pink1* ^−/−^ brain the bioenergetic deficit is clearly more prominent than mitochondrial dynamics failure. Our data that PINK1 deficiency becomes more evident under cell stress might explain the elevated susceptibility of dopaminergic nigrostriatal neurons in PINK1-linked Parkinsonism, since these neurons are known for high levels of oxidative stress from dopamine metabolism. Apparently, mitochondrial dysfunction is present in all tissues with PINK1 deficiency, while additional stress factors explain the tissue specific pattern of neurodegeneration.

### PINK1 deficiency and presynaptic dysfunction

Function in dependence on ATP levels and on membrane potential characterizes mitochondria as well as presynaptic vesicles, where dopamine is imported and stored until exocytotic release. Our findings of reduced dopamine levels in *Pink1^−/−^* striatum at ages between 9 and 24 months extend previous observations, which had reported normal dopamine content but altered release and pathological synaptic plasticity upon electrophysiological analyses of corticostriatal long-term depression and potentiation in *Pink1^−/−^* striatum at ages before 9 months [16]. Since we found no evidence for a loss of dopaminergic terminals in the striatum, a functional rather than structural deficit of presynaptic processes is likely. Whether this relates to our observation of expression changes for the presynaptically localized α-synuclein in *Pink1^−/−^* brain tissue remains unresolved at present. Mitochondrial dysfunction together with α-synuclein expression changes were also documented in fibroblasts from patients with a PINK1 loss-of-function mutation [4], [5], [34]. The gene dosage of α-synuclein is a known modulator of nerve terminal density and function [35], [36]. In view of the early impairment of dopamine release and synaptic plasticity, as well as the subsequent alteration of striatal dopamine levels and the α-synuclein dysregulation before the advent of neurodegeneration in *Pink1^−/−^* mice, we propose that nerve terminal dysfunction underlies the selective deficit in spontaneous locomotor activity.

### The PINK1 deficient mouse as a model of PD neuropathology

The locomotor syndrome displayed by the aged *Pink1^−/−^* mouse is a robust finding, since it was observed in mice with pure 129/SvEv background, through comparison with littermates/closely related control mice, and confirmed in a subsequent progeny cohort. This phenotype is very reminiscent of PD: the progressive reduction of spontaneous movements despite preserved strength and coordination, as well as its manifestation at adult age. In addition, some of the pathophysiological features of PD were also found in PINK1-deficient mice: (i) the reduced dopamine content of the striatum; (ii) the altered expression of α-synuclein mRNA; (iii) a mitochondrial dysfunction that is bound to result in the enhanced oxidative stress susceptibility previously published for *Pink1^−/−^* mice [14]. However, several non-motor signs of early sporadic PD as well as features present in the final stage autopsy tissue such as nigral cell loss and Lewy bodies were not detectable in these mice even after 18 months. Thus, mouse dopaminergic neurons seem to be less susceptible to cell death induced by PINK1-deficiency, when compared with human dopaminergic neurons where PINK1 deficiency was reported to elicit age-related reduction of viability in a cell culture model [18]. Since PARK6 is a rare variant of PD and autopsy nervous tissue from PARK6 patients has not yet become available, the degree of nigral cell loss and Lewy pathology remains unclear for PARK6-linked PD.

In conclusion, we demonstrate for the first time that PINK1 deficiency in mammalian tissue leads to progressive impairment of preprotein import into mitochondria and altered fission-fusion balance under stress, an early phenotype of mitochondrial dysfunction which may well underlie the impaired bioenergetics observed in aged brain tissue. Although neurodegeneration is not detectable within the lifespan of mice and survival remains unchanged, the alteration of dopamine, α-synuclein levels and the novel observation of impaired spontaneous locomotion at old age suggest an accompanying presynaptic pathology which might model early stages of PD.

## Methods

### Generation of *Pink1^−/−^* mice

A mouse genomic bacterial artificial chromosome library constructed from strain 129/SvEv was screened using a cDNA *Pink1* probe. A targeting vector was constructed with a 4.2 kb NheI/NheI fragment containing exon II, III, IV and V as well as a 3.2 kb NheI/BstZ17I fragment. In exon IV, the mutation g/a in position 8343 at position NT_039267 (resulting in the pathogenic G309D mutation) was previously introduced with the QuickChange site directed mutagenesis kit (Stratagene) and primers ctacccagaaggcctggaccacggtcgtacactg and cagtgtacgaccgtggtccaggccttctgggtag (Fig. 1A). Embryonic stem (ES) cell colonies that were resistant both to aminoglycoside G418 and 2′-fluoro-2′deoxy-1-β-D-arabinofuranosyl-5-iodouracil (FIAU) were screened with Southern blotting (AccI digestion and outside probe) to identify correctly targeted cell clones (Fig. 1B) and the correctly targeted clones used for blastocyst injections. F1 heterozygous animals with a pure inbred 129/SvEv background were used to derive homozygous mutant and wildtype mouse lines maintained through breeding among homozygotes. Heterozygous animals were not analyzed further, in view of cage space limitations and in order to reduce genotyping efforts to PCR. Animal husbandry guidelines from the German Animal Welfare Act, the Council Directive of 24 November 1986 (86/609/EWG) with Annex II and the ETS123 (European Convention for the Protection of Vertebrate Animals) were followed, keeping the lines at the FELASA-certified Central Animal Facility (ZFE) of Frankfurt University Medical School. Genomic DNA was isolated from tail biopsies and routine genotyping occurred with the following three different PCR amplifications (for primer sequences compare Table S1): (i) demonstration of the point mutation using PCR primers exon 4 mouse F/exon 4 mouse R to generate a DNA amplicon of 464 bp and a subsequent AvaII restriction digest to fragments of 288 bp/176 bp in wildtype versus 176 bp/154 bp/134 bp in the homozygotes, (ii) detection of the *Neo* selection marker with PCR primers *Pink1*intron5F/*Neo*R, (iii) deletion detection with PCR primers *Pink1*intron5F/*Pink1*intron5R. RNA was isolated from brain, liver and skeletal muscle with Trizol reagent (Invitrogen) to test expression. cDNA synthesis was carried out with SuperScript III (Invitrogen), the PCR of individual *Pink1* mRNA exon boundaries was performed with AmpliTaq (AppliedBiosystems) using the primers detailed in Table S1. The quantitative real-time RT-PCR was done with TaqMan gene expression assays Mm00550823_m1 and Mm00550827_m1.

### Behaviour and motor function

All tests were performed in an initial cohort of 16 (7 females and 9 males) homozygous *Pink1^−/−^* and 16 wildtype (6 females and 10 males) mice at 4, 10 and 16 months of age. General health and neurological performance were evaluated with the SHIRPA test [37]. The weight as a measure of metabolism, nest-building as a measure of normal development, and the production of urine/faeces during 5 minutes open field/5 minutes Rota-Rod tests together with center/margin time during open field test as measures of anxiety were observed and recorded. To assay strength and coordination, grip-strength, inverted-screen, pole-test, Rota-Rod and foot-print performance were analyzed without previous training as described previously [38]. The spontaneous movement of mice in a 20×20 cm arena was registered by infra-red beams in a Digiscan monitor (AccuScan, Columbus, OH) [38]. Open field spontaneous movement was reassessed in a second cohort of homozygous progeny at the ages 9 and 24 months, the same animals which were later sacrificed for dopamine neurochemistry. Hyperhidrosis assays were performed as published [39], [40]. Acoustic startle tests were carried out as described previously [21].

### Histological analysis of brain tissue

Two 16 month old brains from *Pink1^−/−^* and wildtype mice were fixed with 4% paraformaldehyde, embedded in Paraplast-plus and cut in 5 µm thick coronal consecutive sections from the brainstem to the striatum. The first of every five sections was subjected to Nissl staining of neurons and Klüver-Barrera counterstaining of myelinated nerve fiber tracts.

### Immunohistochemistry

The second of every five sections was immersed in 3% H_2_O_2_, 0.05 M Tris, 0.9% NaCl, pH 7.5 for 30 minutes, washed and immersed for 60 minutes in 0.25% Triton ×100, 5% BSA and 0.1 M DL-lysine for α-synuclein immunohistochemistry. Sections were incubated overnight in the first antibody (anti-α-synuclein from Transduction labs at 1∶200, anti-TH from Pel Freeze at 1∶1000, GFAP from Sigma at 1∶500) at room temperature. On the next day after rinsing, sections were incubated for one hour at room temperature with the appropriate secondary biotinylated antibody. Following washes, sections were incubated for 1 hour with the avidin-biotin-peroxidase complex (1∶100, ABC-Elite, Vector Laboratories). Following several washes, the sections were reacted in a 3,3-diaminobenzidine tetrahydrochloride solution (0.07% DAB and 0.002% H_2_O_2_). The sections were dehydrated and mounted.

### Stereological analysis and Optical density

Non-biased stereological analysis of TH-positive somata in complete serial sections of the midbrain using the Stereoinvestigator Software package (MFB Bioscience) and optical density analysis of striatal TH immunoreactivity were performed as described previously [41].

### Dopamine levels in HPLC

The striatum was homogenized in 20–40 volumes of cold 150 mM potassium phosphate buffer, pH 6.8. Each homogenate was aliquoted to analyze the protein concentration according to Lowry and analyze the dopamine content in parallel, using high performance liquid chromatography (HPLC) coupled to electrochemical detection, following a previously pulished method [42]: One aliquot was diluted (½) with 0.4 N perchloric acid containing 0.4 mM sodium disulfite, 0.90 mM EDTA and the corresponding internal standard (dihydroxybenzylamine for catecholamines). Afterwards, samples were centrifuged for 3 min (15000 *g*) and the supernatants directly injected into the HPLC system. The HPLC system consisted of the following elements: The pump was an isocratic Spectra-Physics 8810. The column was a RP-18 (Spherisorb ODS-2; 150 mm, 4.6 mm, 5 µm particle size; Waters, Massachusetts, USA). The mobile phase, previously filtered and degassed, consisted of 100 mM citric acid, 100 mM sodium acetate, 1.2 mM heptane sulphonate, 1 mM EDTA and 7% methanol (pH 3.9). The flow rate was 0.8 ml/min. The effluent was monitored with a coulochemical detector (Coulochem II, ESA) using a procedure of oxidation/reduction (conditioning cell: +360 mV; analytical cell #1: +50 mV; analytical cell #2: −340 mV), a procedure that reaches a sensitivity of 50 nA (10 pg *per* sample). The signal was recorded on a Spectra-Physics 4290 integrator. The results were obtained from the peaks and calculated by comparison with the area under the corresponding internal standard peak. Values were expressed as ng/mg of protein.

### Immunoblots

Protein extraction and immunoblotting were performed as described previously [4], with primary antibody titers as follows: anti-α-synuclein (1∶500, BD Transduction Laboratories), anti-β-actin (1∶10000, Sigma), anti-tyrosin-hydroxylase (1∶1000, Pel Freez). For densitometry, the Image Master Total Lab software (Amersham Pharmacia) was used. As protein markers of inner and outer mitochondrial membranes, MitoProfile Rodent Total OXPHOS complexes (Mitosciences) and Porin (Calbiochem) antibodies were used. Formation of OPA1 isoforms in total cell extracts from heart, brain (frontal cortex), and liver tissues was analyzed by immunoblotting using affinity-purified antibodies raised against the C-terminus of OPA1 [43].

### Transcript levels

18 months old mice brains were dissected to use one hemisphere for RNA, the other hemisphere for protein extraction. Cerebellum, brain stem, midbrain and cortex were extracted separately with Trizol (Invitrogen) following manufacturer's instructions. The RNA from striatum was extracted with RNeasy Lipid tissue mini kit from Qiagen. Total RNA was digested with DNAse amplification grade (Invitrogen), one microgram was reverse transcribed using pd(N)_6_ and NotI d(T)_18_ primers and the High Capacity kit (Applied Biosystems), and the qPCR was carried out in 20 µl on an ABI Prism 5700 sequence detection system. The changes of transcripts (qPCR Taqman® assays Mm00447331_m1 for *Snca* or a SYBRGreen assay with primers 5′-TGGCAGTGAGGCTTATGAAA-3′ and 5′-ATGACTGGGCACATTGGAAC-3′) were validated after independent cDNA synthesis with SuperScript III Reverse Transcriptase in a qPCR with *Ywhaz* (Mm01158417_g1) and *Gapdh* (Mm99999915_g1) as internal control. Expression changes were analyzed both with the 2^−ΔΔCt^ method.

### Mitochondrial membrane potential (Δψm) and ATP level

Dissociated brain cells were prepared as described [44]. To measure Δψm, the cells were plated on a 24 well plate and incubated for 15 min with 0.4 mM of the fluorescence dye rhodamine 123 (R123). ATP levels in dissociated brain cells were determined using the bioluminescent measurement of ATP as published [45]. The preparation of isolated mitochondria was conducted as published [46], measuring Δψm with R123 at a final concentration of 0.4 µM with the Victor multiplate reader (Perkin Elmer Life Sciences) at 490 nm (excitation)/535 nm (emission). The data (n = 8) are expressed at fluorescence units per mg/ml protein. The effects were robust across independent preparations, irrespective of the percentage of glia admixture to neuronal cells in culture.

### Respirometry

The whole brains of twenty two mice aged to 21–23 months were rapidly removed, washed and placed in ice-cold buffer containing 225 mM mannitol, 75 mM sucrose, 5 mM HEPES, BSA (1 mg/ml), 1 mM EGTA, pH 7.4. The tissue was then minced with scissors and placed in 10 ml of isolation medium supplemented with nagarase (0.5 mg/ml) and then homogenized with the use of motor-driven Teflon-glass Potter homogenizer. After two time dilution, the homogenate was then centrifuged at 2000×g for 9 min. The supernatant was decanted and centrifuged at 12000×g for 11 min. To permeabilize synaptosomes, the pellet was suspended in isolation buffer supplemented with digitonin (0.2 mg/ml) and homogenized manually. Finally the suspension was centrifuged at 12000×g for 11 min. The mitochondrial pellet was resuspended in isolating medium at a protein concentration of 20–40 mg/ml. All procedures were carried out at 4°C. Mitochondrial oxygen consumption was measured at 30°C using an Oroboros oxygraph (Anton Paar, Austria) in a medium containing 10 mM KH_2_PO_4_, 60 mM KCl, 60 mM Tris, 5 mM MgCl_2_, 110 mannitol and 0.5 mM EDTA-Na_2_, pH 7.4. 10 mM glutamate+5 mM malate were used as respiratory substrates for complexes I+III+IV (Cx I) and 500 µM TMPD (*N*,*N*,*N′*,*N′*-tetramethyl-*p*-phenylendiamine)+1 mM ascorbate as respiratory substrates for complex IV (Cx IV). The concentration of mitochondria was 0.3 mg protein/ml.

### Mitochondrial import

Isolation of mitochondria from mouse liver and import of proteins was performed essentially as described [47]: In brief, the mouse liver was homogenized in ice-cold solution A (0.22 M mannitol, 0.07 M sucrose, 0.02 M HEPES, pH 7.6, 1 mM EDTA, 0.1% BSA, 1 mM PMSF). Cell debris was removed by centrifugation at 1,500 *g* for 5 min. Mitochondria were pelleted by centrifugation at 10,000 *g* for 10 min and resuspended in solution B (0.22 M mannitol, 0.07 M sucrose, 0.02 M HEPES, pH 7.6, 1 mM EDTA, 1 mM PMSF). Both centrifugation steps were repeated three times. Radiolabelled pre-ornithine-transcarbamylase (pOTC) was generated by *in vitro* transcription and translation in rabbit reticulocyte lysate and imported into isolated mitochondria after dilution in import buffer (20 mM HEPES-KOH, pH 7.6, 250 mM sucrose, 5 mM MgAc, 160 mM KAc, 5 mM succinate, 5 mM malate, 1 mM DTT) to a final concentration of 125 to 250 µg/ml at 30°C in the presence of 3 mM ATP. In a control sample, the membrane potential (Δψm) was dissipated by addition of 8 µM antimycin A, 0.5 µM valinomycin, and 2 µM oligomycin. Non-imported preproteins were removed by trypsin treatment. Samples were analyzed by SDS-PAGE and autoradiography. Imported proteins were quantified using the ImageQuant software (GE Healthcare). Isolation of mitochondria from cultured fibroblasts was performed by homogenization of cells in solution A and differential centrifugation as described [48].

### Mitochondrial morphology

For fluorescent microscopic analysis, cortical neurons were isolated from 4 day old wildtype or *Pink1* ^−/−^ mice as published previously [49] and cultivated on cover slides. Cells were stained with Mitotracker Red 14 d after isolation and fixed. Mitochondrial morphology was divided into 4 morphotypes and quantified by blinded investigators. To induce proteasomal stress, cells 13 d after isolation were treated with 10 µM MG132 (Calbiochem) for 24 h before fixation. n = 3 for each condition, with at least 100 cells/experiment. For electron microscopy, 4 brains from 22–24 months old mice were fixed by transcardial perfusion with saline, followed by perfusion and immersion using 2% paraformaldehyde (PFA), cut into small blocks, which were postfixed with 4% PFA and 0.1% glutaraldehyde on ice for 2 h. After being washed twice in PBS with 0.02% glycine, the small blocks were infused with 2.3 M sucrose overnight, mounted on small metal pins, frozen and stored in liquid nitrogen. Ultrathin sections were cut at −110°C using a diamond knife (Diatome) in an ultracryomicrotome (Leica), collected using a 1∶1 mixture of 1.8% and 2.3 M sucrose and deposited on formvar- and carbon-coated grids. Sections were contrasted with uranyl acetate methyl cellulose on ice for 10 min, embedded in the same solution and examined with a Phillips CM120 electron microscope.

### Statistics

The statistical evaluation of data by unpaired two-tailed t-test or survival plots and their graphical documentation for figures was carried out with the GraphPad software. Significance was indicated by asterisks (* for p<0.05; ** for p<0.01; *** for p<0.001).

## Supporting Information

Table S1 Primer sequence information.
(0.03 MB DOC)

Figure S1 Lack of peripheral paralysis. Normal motor performance of Pink1−/− (n = 16) mice at age 16 months in grip strength tests indicated intact muscular and peripheral nervous system.
(2.55 MB TIF)

Figure S2 No deficit of coordination in Rota-Rod tests. The ability to maintain equilibrium on a rotating rod was similar for naïve Pink1−/− (n = 16) and wildtype (n = 16) mice at 16 months of age.
(1.70 MB TIF)

Figure S3 Normal measures of anxiety in Pink1−/− mice. The quantification of margin time versus center time in open field tests, which correlate to exploration versus anxiety, did not show a significant difference in 16 months old animals (n = 16 for each group). Furthermore, the counting of fecal boli deposited showed no abnormality.
(1.17 MB TIF)

Figure S4 Normal lifespan of Pink1−/− mice. A survival curve of 55 Pink1−/− versus 46 wildtype mice showed no significant differences.
(2.72 MB TIF)

Figure S5 Lack of autonomic dysfunction in hyperhidrosis assays. Provocation of hyperhidrosis with pilocarpine showed similar responses in two different assays for Pink1−/− (n = 11) and wildtype (n = 11) mice at 18 months of age.
(1.46 MB TIF)

Figure S6 Lack of brainstem pathology in acoustic startle tests. The quantification of startle responses of 8–10 (n = 8 for each group) and 24 months old (n = 8 for each group) Pink1−/− mice (KO) to acoustic stimuli varying from 90 to 120 decibels (db) failed to detect a significant difference from wildtype values (WT), indicating normal function of noradrenergic neuron pathways in the locus coeruleus.
(1.88 MB TIF)

Figure S7 Lack of Lewy bodies in Pink1−/− brain. Immunohistochemical analyses did not detect the round or elongated aggregates of α-synuclein within the neuronal cytoplasm, which characterize Lewy pathology in PD affected cells. However, enhanced α-synuclein immunoreactivity (brown color, with blue hematoxylin counterstain) throughout the lower Pink1−/− (KO) brainstem with somatodendritic distribution in the dorsal motor vagal nucleus (nucl. X) (Cbll. = cerebellum, Area Post. = area postrema, Sp. C. = spinal canal, insets shown below in higher magnification) was apparent in independent stains of various Pink1−/− mice (B, D), but not wildtype mice (A, C).
(2.01 MB TIF)

Figure S8 Reduced expression of α-synuclein mRNA in Pink1−/− brain. Dissected brain areas from Pink1−/− (n = 5) and wildtype (n = 5) 24 months old PINK1-deficient mice were extracted for protein and mRNA. Normalized levels of alpha-synuclein (SNCA) in brainstem and midbrain consistently showed an increase for the protein, which missed significance, but a significant decrease for the corresponding mRNA.
(1.15 MB TIF)

Figure S9 Unchanged OPA1 isoforms in Pink1−/− tissue at age 18 months. Total cell extracts of heart, brain (frontal cortex), and liver tissues of wild type (WT) and Pink1−/− (KO) mice were generated and equal amounts of total protein were analyzed by SDS-PAGE and immunoblotting using antibodies raised against the C-terminus of OPA1. Formation of OPA1 isoforms is not affected by the presence of PINK1. OPA1 isoforms (L1, L2, S3, S4, S5) and putative fragments (f, f′) are indicated.
(1.90 MB TIF)
