# Supplementary material for: Parkinson Phenotype in Aged PINK1-Deficient Mice Is Accompanied by Progressive Mitochondrial Dysfunction in Absence of Neurodegeneration
Source: PLoS One. 2009 Jun 3;4(6):e5777. doi: 10.1371/journal.pone.0005777 (PMC2686165; doi:10.1371/journal.pone.0005777)
Supplement: Table S1 — Primer sequence information. (0.03 MB DOC) [file pone.0005777.s001.doc]

G309D-PINK1 mutation detection:

exon 4 mouse F: 5’ GGAGAAGTCACCCCTGTTGG 3’

exon 4 mouse R: 5’ ctctcatttctgcgtgctttgttc 3’

Routine mouse genotyping:

*Pink1* intron 5F: 5’ AGTGCTCCAAGAAGGGGCATTAGA 3’

*Pink1* intron 5R: 5’ GCTGGGGAGATTGCTCAGTGGTTA 3’

neoR (*Pink1* construct): 5’ GATCAGCAGCCTCTGTTCCACATA 3’

Splicing analysis:

*Pink1* exon 1F: 5’ GGCTCCGGTCACCGCGCCACCAT 3’

*Pink1* exon 2R: 5’ ATGGCAAAGGGAAAGGTGGGAGTC 3’

*Pink1* exon 2F: 5’ AGAAAACCAAGCGCGTGTCTGACC 3’

*Pink1* exon 3R: 5’ GTAACTGCTCCATACTCTCCAG 3’

*Pink1* exon 3F: 5’ AGGTTCCTCCAGCGAAGCCATCTT 3’

*Pink1* exon 4R: 5’ TCTTCATAACGAGGAACAGTGTGC 3’

*Pink1* exon 4F: 5’ CCCTAACATCATCCGGGTTTTC 3’

*Pink1* exon 5R: 5’ CAAGGATGTTGTCGGACTTGAGAT 3’

*Pink1* exon 5F: 5’ CCTGCGCCAGTACCTTGAGGAG 3’

*Pink1* exon 6R: 5’ GGGCCATCAGGGAGCCATTG 3’

*Pink1* exon 6F: 5’ CCCTGGCTAGTGATCTCAGACTTT 3’

*Pink1* exon 7R: 5’ CTGGCCTCTCGCTGGAGCAGTG 3’

*Pink1* exon 7F: 5’ CATTCTGGCCCCAGTGCGGTAAT 3’

*Pink1* exon 8R: 5’ GGCCCTCTATAGCTTACCAACTCC 3’
