# Supplementary figures and images for: Parkinson Phenotype in Aged PINK1-Deficient Mice Is Accompanied by Progressive Mitochondrial Dysfunction in Absence of Neurodegeneration
Source: PLoS One. 2009 Jun 3;4(6):e5777. doi: 10.1371/journal.pone.0005777 (PMC2686165; doi:10.1371/journal.pone.0005777)

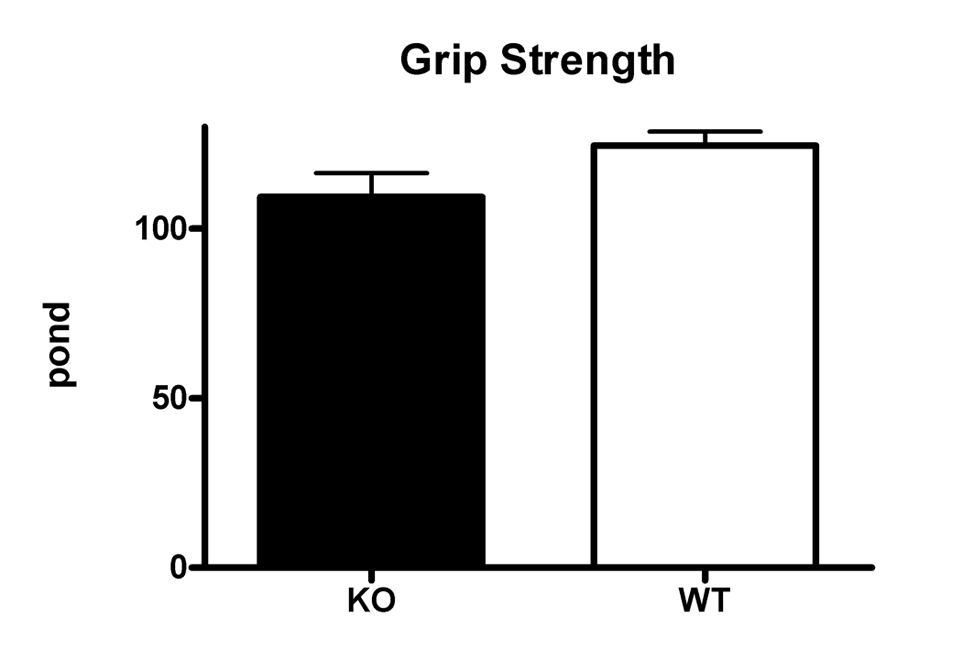

Supplement: Figure S1 — Lack of peripheral paralysis. Normal motor performance of Pink1−/− (n = 16) mice at age 16 months in grip strength tests indicated intact muscular and peripheral nervous system. (2.55 MB TIF) [file pone.0005777.s002.tif]

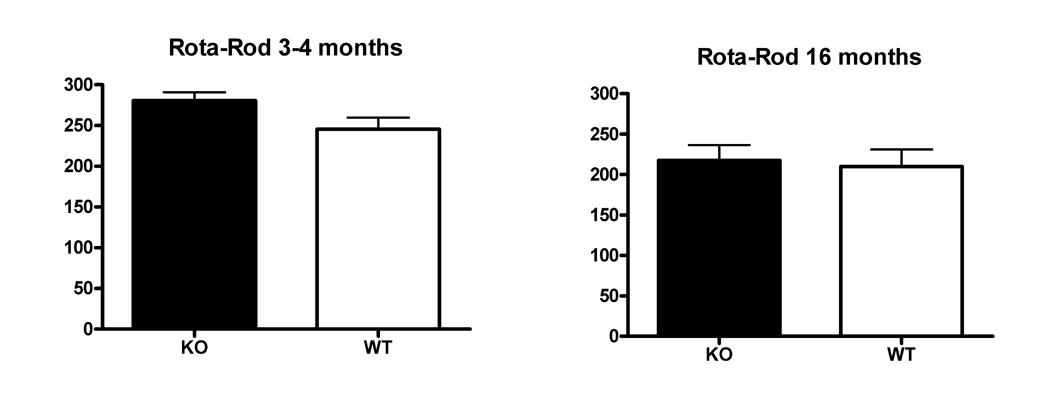

Supplement: Figure S2 — No deficit of coordination in Rota-Rod tests. The ability to maintain equilibrium on a rotating rod was similar for naïve Pink1−/− (n = 16) and wildtype (n = 16) mice at 16 months of age. (1.70 MB TIF) [file pone.0005777.s003.tif]

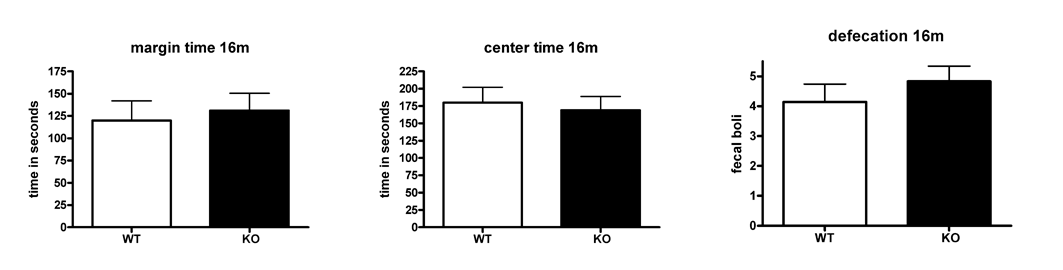

Supplement: Figure S3 — Normal measures of anxiety in Pink1−/− mice. The quantification of margin time versus center time in open field tests, which correlate to exploration versus anxiety, did not show a significant difference in 16 months old animals (n = 16 for each group). Furthermore, the counting of fecal boli deposited showed no abnormality. (1.17 MB TIF) [file pone.0005777.s004.tif]

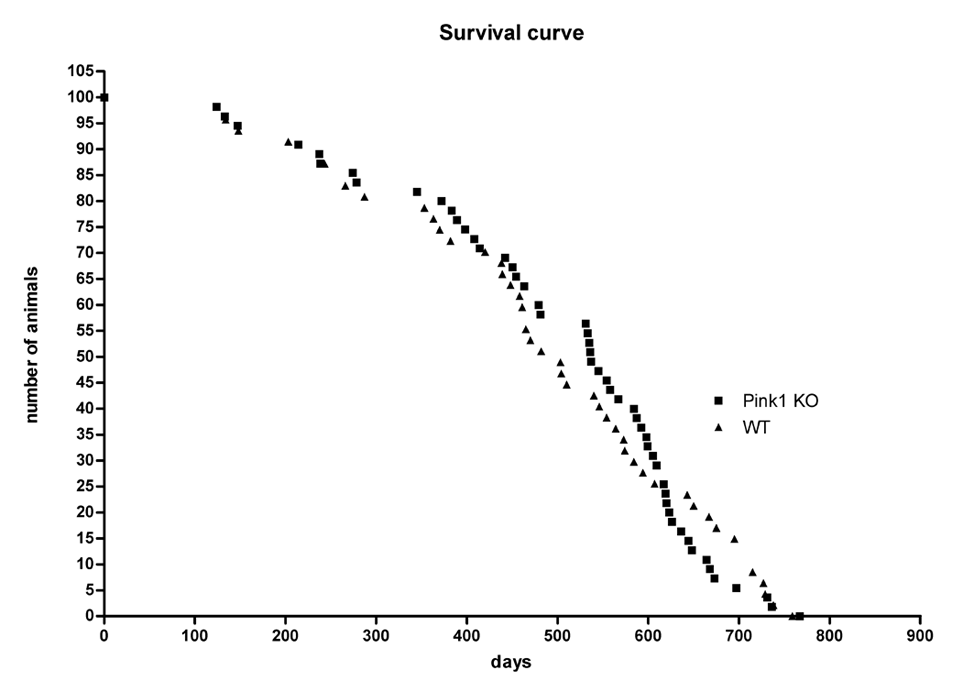

Supplement: Figure S4 — Normal lifespan of Pink1−/− mice. A survival curve of 55 Pink1−/− versus 46 wildtype mice showed no significant differences. (2.72 MB TIF) [file pone.0005777.s005.tif]

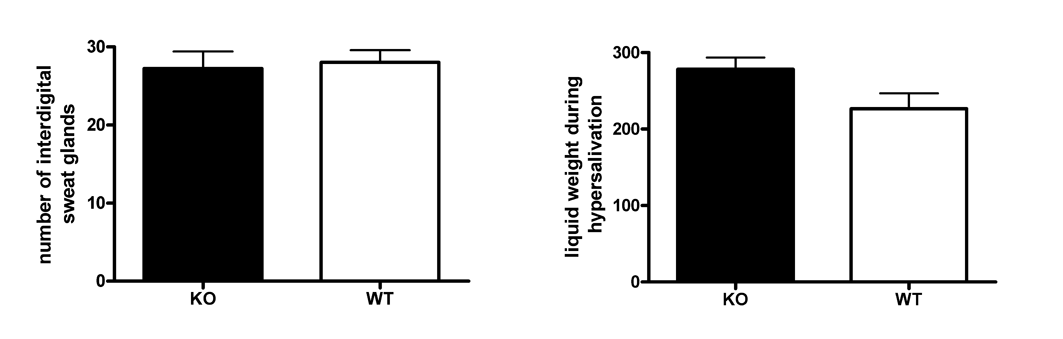

Supplement: Figure S5 — Lack of autonomic dysfunction in hyperhidrosis assays. Provocation of hyperhidrosis with pilocarpine showed similar responses in two different assays for Pink1−/− (n = 11) and wildtype (n = 11) mice at 18 months of age. (1.46 MB TIF) [file pone.0005777.s006.tif]

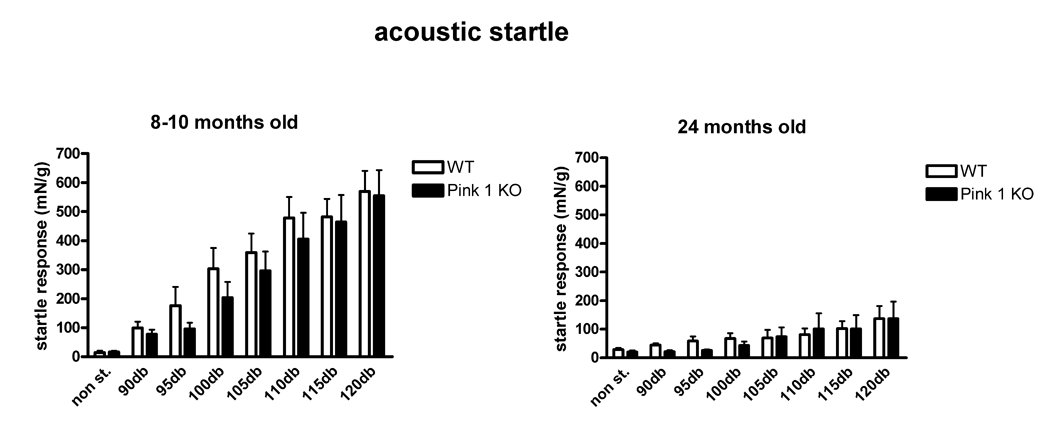

Supplement: Figure S6 — Lack of brainstem pathology in acoustic startle tests. The quantification of startle responses of 8–10 (n = 8 for each group) and 24 months old (n = 8 for each group) Pink1−/− mice (KO) to acoustic stimuli varying from 90 to 120 decibels (db) failed to detect a significant difference from wildtype values (WT), indicating normal function of noradrenergic neuron pathways in the locus coeruleus. (1.88 MB TIF) [file pone.0005777.s007.tif]

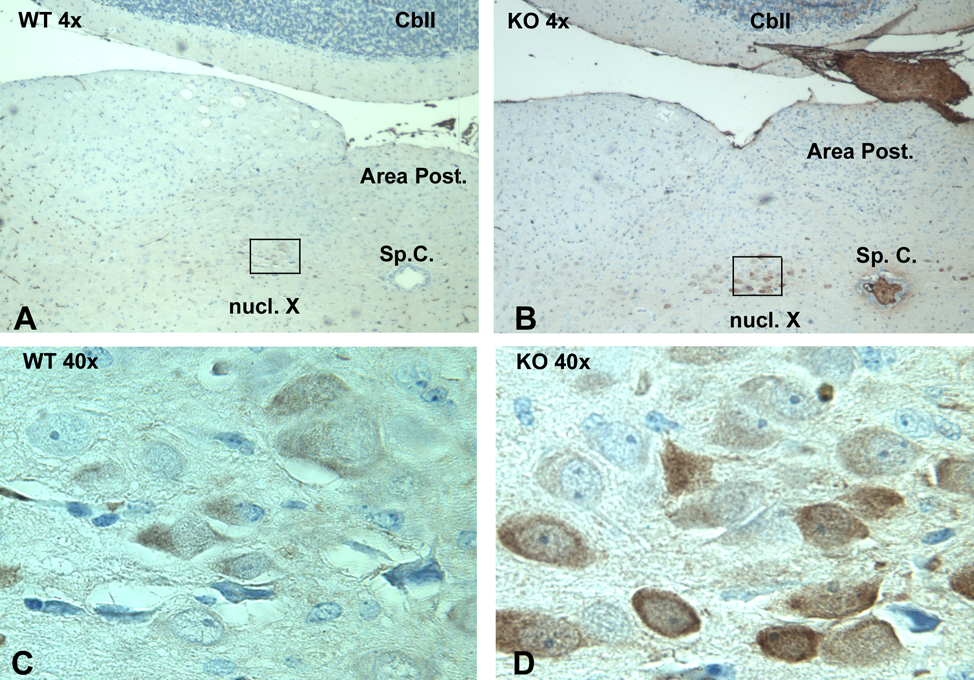

Supplement: Figure S7 — Lack of Lewy bodies in Pink1−/− brain. Immunohistochemical analyses did not detect the round or elongated aggregates of α-synuclein within the neuronal cytoplasm, which characterize Lewy pathology in PD affected cells. However, enhanced α-synuclein immunoreactivity (brown color, with blue hematoxylin counterstain) throughout the lower Pink1−/− (KO) brainstem with somatodendritic distribution in the dorsal motor vagal nucleus (nucl. X) (Cbll. = cerebellum, Area Post. = area postrema, Sp. C. = spinal canal, insets shown below in higher magnification) was apparent in independent stains of various Pink1−/− mice (B, D), but not wildtype mice (A, C). (2.01 MB TIF) [file pone.0005777.s008.tif]

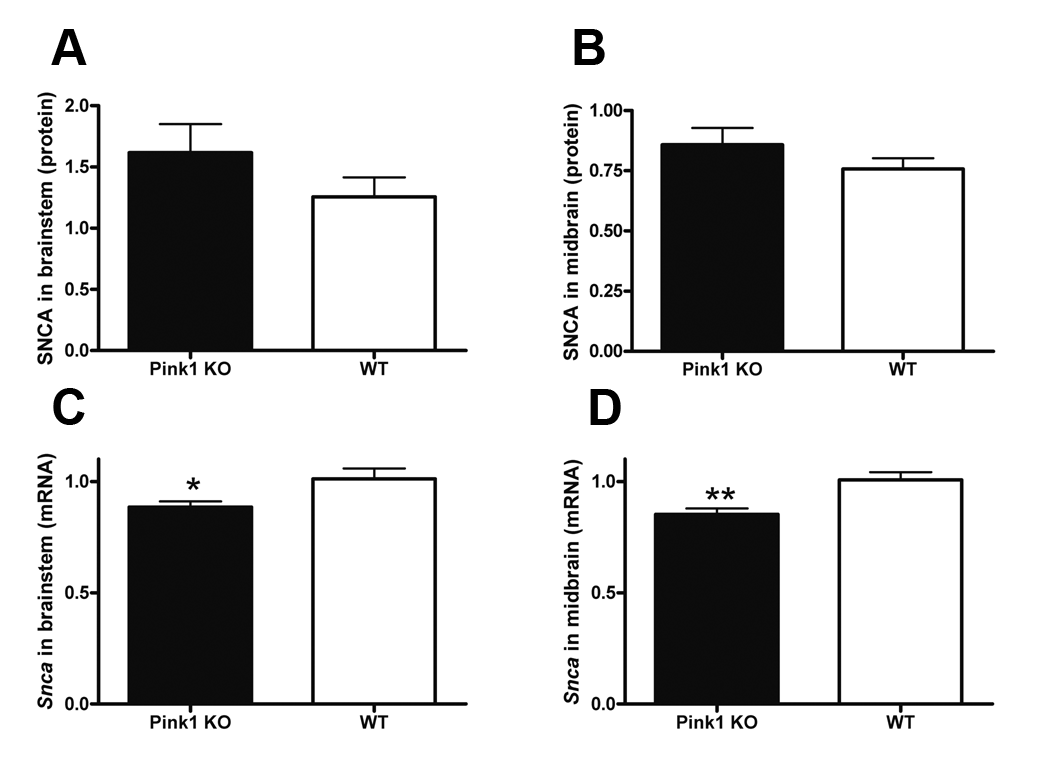

Supplement: Figure S8 — Reduced expression of α-synuclein mRNA in Pink1−/− brain. Dissected brain areas from Pink1−/− (n = 5) and wildtype (n = 5) 24 months old PINK1-deficient mice were extracted for protein and mRNA. Normalized levels of alpha-synuclein (SNCA) in brainstem and midbrain consistently showed an increase for the protein, which missed significance, but a significant decrease for the corresponding mRNA. (1.15 MB TIF) [file pone.0005777.s009.tif]

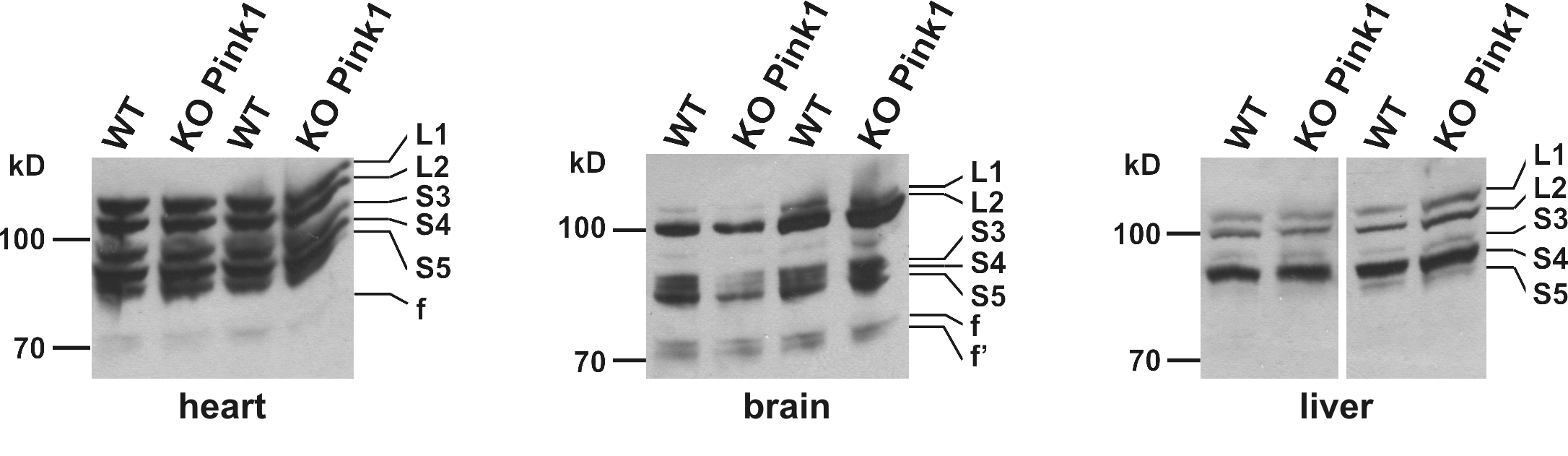

Supplement: Figure S9 — Unchanged OPA1 isoforms in Pink1−/− tissue at age 18 months. Total cell extracts of heart, brain (frontal cortex), and liver tissues of wild type (WT) and Pink1−/− (KO) mice were generated and equal amounts of total protein were analyzed by SDS-PAGE and immunoblotting using antibodies raised against the C-terminus of OPA1. Formation of OPA1 isoforms is not affected by the presence of PINK1. OPA1 isoforms (L1, L2, S3, S4, S5) and putative fragments (f, f′) are indicated. (1.90 MB TIF) [file pone.0005777.s010.tif]
